# Phytochemistry, Toxicology and Therapeutic Value of *Petasites hybridus* Subsp. Ochroleucus (Common Butterbur) from the Balkans

**DOI:** 10.3390/plants9060700

**Published:** 2020-05-31

**Authors:** Tatjana Mihajilov-Krstev, Boris Jovanović, Bojan Zlatković, Jelena Matejić, Jelena Vitorović, Vladimir Cvetković, Budimir Ilić, Ljubiša Đorđević, Nataša Joković, Dragoljub Miladinović, Tatjana Jakšić, Nemanja Stanković, Vesna Stankov Jovanović, Nirit Bernstein

**Affiliations:** 1 Department of Biology and Ecology, Faculty of Sciences and Mathematics, University of Niš, 18000 Niš, Serbia; tatjanamk69@gmail.com (T.M.-K.); bojanzlat@yahoo.com (B.Z.); jelena.rajkovic@gmail.com (J.V.); biovlada@yahoo.com (V.C.); ljupce@pmf.ni.ac.rs (L.Đ.); natasa.jokovic@pmf.edu.rs (N.J.); 2 Department of Natural Resource Ecology and Management, Iowa State University, Ames, IA 50011, USA; nanoaquatox@gmail.com; 3 Faculty of Medicine, University of Niš, 18000 Niš, Serbia; jekamatejic@gmail.com (J.M.); bucabule@yahoo.com (B.I.); dragoljubm@gmail.com (D.M.); 4 Department of Biology, Faculty of Sciences and Mathematics, University of Priština, 38220 Kosovska Mitrovica, Serbia; tatjana.jaksic@pr.ac.rs; 5 Institute of Public Health, 18000 Niš, Serbia; nemanjastanko@yahoo.com; 6 Department of Chemistry, Faculty of Sciences and Mathematics, University of Niš, 18000 Niš, Serbia; sjvesna@pmf.ni.ac.rs; 7 Institute of Soil Water and Environmental Sciences, Volcani Center 15159, Israel

**Keywords:** *Petasites*, medicinal plant, essential oil, sesquiterpenes, pyrrolizidine alkaloids, toxicity, Balkan, anti-inflammatory, skin irritation, insect repellent, anti cholinesterase

## Abstract

*Petasites hybridus* (Common butterbur) is extensively used in traditional medicine, and is currently gaining interest and popularity as a food supplement and for its medicinal properties. It contains a large number of active compounds of potential therapeutic activity, but also toxic pyrrolizidine alkaloids. Science-based information is needed to support the developing modern use of this plant, and to direct continued safe practice in traditional medicine. The present study focused on the essential oils from leaves and rhizomes of the understudied *P. hybridus* ssp. ochroleucus from the Balkans, and evaluated its phytochemistry and potential therapeutic activities (antimicrobial, antioxidant, anti-cholinesterase and anti-inflammatory), as well its toxicology potential (acute toxicity in insects and mice). We studied the essential oils, which are not commonly used in traditional practices, but have a potential for safe use since the toxic pyrrolizidine alkaloids, which are non-volatiles, are usually not present in the distilled essential oils. Pyrrolizidine alkaloids were indeed not detected in the essential oils; ingestion of the essential oils did not induce toxicity signs in mice, and topical application did not elicit skin irritation in humans. The essential oils had no antimicrobial properties against 20 pathogenic bacterial strains, but demonstrated good local anti-inflammatory activity in a Carrageenan-induced paw edema test. An insect toxicity test demonstrated that the leaf essential oil is an efficient insect repellent, and the demonstrated anti-cholinesterase activity suggests a potential for the treatment of neurological conditions. Isopetasin, a sesquiterpene found in plants of the genus *Petasites*, known to have anti-inflammatory effects, was present only in the rhizomes essential oil (3.9%), and sesquiterpene lactones concentrations were high, likely contributing to the antioxidant activity.

## 1. Introduction

Common butterbur [*Petasites hybridus* (L.) G.Gaertn., B.Mey. & Scherb] is a perennial herbaceous flowering plant of the daisy (Asteraceae) family. It has long rhizomes and large leaves, which develop after flowering and can reach up to 60 cm in diameter, and it grows up to 3 feet in height and has a history of use in traditional medicine [1]. It is native to Europe, and is present as an introduced species in North America and West and North Asia. It is common on riverbanks, in wet meadows and in other damp and shady locations [2]. Herbal preparations of *P. hybridus* have been practiced in traditional medicine in Europe for over 900 years, for the treatment of a broad spectrum of human aliments [3,4]. The rhizomes with roots (*Rhizoma cum radicibus Petasiti*) and the leaves (*Folium Petasiti*) are used to treat spastic cough, bronchitis, allergic rhinitis, asthma, migraine, dysmenorrhea, hypertension, ulcers, loss of appetite, as well as inflammatory gastrointestinal and genitourinary diseases [4,5,6,7,8]. In addition to its traditional use, *P. hybridus* is increasingly used today for its medicinal properties as an alternative medicine [9], and is rapidly gaining popularity as a dietary supplement [4]. Commercial preparations of *P. hybridus* capsules, extracts, powders, tinctures and softgels are available nowadays in many countries around the world. 

Although the extracts of *P. hybridus* are approved by the USA Food and Drug Administration for use as dietary supplement, negative side effects (e.g., gastrointestinal problems, nausea, headache, drowsiness and halitosis) are known to occur [4]. Toxic effects are mainly attributed to alkaloid compounds—pyrrolizidine alkaloids, which are known to cause liver damage and may induce cancer [10]—and it is therefore not recommended for self-medication [11]. The concentrations of pyrrolizidine alkaloids in commercial preparations are usually below the limit of detection due to the production processes [4]. However, traditional processing, which is exercised in the Balkan Peninsula, results in potential exposure of the users to high concentrations of pyrrolizidine alkaloids. Following the Balkan’s ethnobotanical tradition, preparations of *P. hybridus* are widespread for the treatment of gastrointestinal and parasitic-induced hepatobiliary and respiratory disorders, as well as migraine and tension headaches [12,13,14,15]. 

The present study focused on *Petasites hybridus* subsp. ochroleucus, (i.e., a subspecies of *Petasites hybridus*) which is spread and used in traditional medicine mainly in the eastern, central and southern parts of the Balkans [1,16], and is endemic to the southern Balkan region. Very little information is available on this sub-species, which is distinguished from the more common and widely distributed, red-flowered subspecies *P. hybridus* subsp. hybridus, which is common in the northern and western parts of the Balkans, as well as in the rest of Europe. We focused on the essential oil of this plant, which is a herbal preparation not commonly used in traditional practices of *Petasites hybridus* and therefore has attracted little research-attention so far, but that has a potential for safe use since the toxic pyrrolizidine alkaloids are non-volatiles and are therefore usually not present in essential oils. 

To the best of our knowledge, the chemical composition of the essential oil of *P. hybridus*, subsp. ochroleucus has not been studied before, and only limited information is available regarding its biological activities, despite the common use of the plant by the people of the Balkans. Variability in chemical composition within and between *P. hybridus* species is known to occur [11,17]. We therefore analyzed the chemical profile of the essential oils, as well as their effect on a range of biological and toxicological activities. Chemical composition may also vary between plant organs, and we have therefore comparatively evaluated the composition and activities of essential oils from leaves and rhizomes. 

External application of *P. hybridus* is uncommon, and it could potentially be a safer mode of application. There are indications for external use of *P. hybridus* in the western Balkan Peninsula, for the treatment of rheumatism and musculoskeletal ailments and pains [14]. Additionally, according to reports in the 19th century literature from Serbia, it was well known and respected as an external remedy, with anti-inflammatory and wound healing properties, and as an effective treatment for swellings and skin ulcers [18]. We have therefore also evaluated the skin irritant potential of topical applications of the essential oil.

The overall aim of this project was to evaluate therapeutic and toxicology activities of the essential oil of *P. hybridus* subsp. ochroleucus. The hypotheses guiding the workplan were: (1) Essential oils from various plant parts vary in chemical composition and hence in therapeutic and toxicological effects. (2) The essential oils will not cause skin irritation when applied externally. To evaluate these hypotheses, we studied: (A) Pytochemistry (chemical profile) of essential oils from the leaves (above ground tissue) and rhizomes (below ground tissue) of *P. hybridus* subsp. ochroleucus. (B) Antimicrobial, antioxidant, anti-inflammatory and anti-cholinesterase activities, as well as toxicological effects (in mouse and Drosophila), comparatively, for essential oils from the leaves and rhizomes. (C) Skin irritation potential in humans, with external application of the leaf and rhizome essential oils. Antimicrobial activity was evaluated against 20 pathogenic bacterial strains (10 laboratory reference strains and 10 strains isolated from human wound swabs). Anti-inflammatory activity was studied in a Carrageenan-induced paw edema test with female Sprague Dawley rats.

## 2. Results

The chemical profiles of the essential oils of *P. hybridus* subsp. ochroleucus are presented in Table 1. Essential oil from the leaves [yield = 0.015% (*v/w*), d = 848.0 µg/µL] contained 42 components (94.62% of the total oil). Only five compounds had content higher than 3%. These are, in decreasing order: Fukinanolide (33.42%), 7-epi-α-Eudesmol (16.14%), Linalool (9.03%), Eremophilene (4.31%) and Geramacrene D (4.26%). Essential oil from the rhizome [yield = 0.067% (*v/w*), d = 914.0 µg/µL] contained 60 components (92.97% of total oil). Nine compounds had content higher than 3%. These are, in decreasing order: (2E)-Nonenal (11.23%), 1-Nonene (8.57%), Germacrene D (5.01%), α-Eudesmol (4.52%), Isopetasin (3.93%), α-Bisabolol oxide (3.38%), epi-β-Santalene (3.45%), α-Santalene (3.36%) and β-Cubebene (3.13%).

Essential oils from both leaves and the rhizomes of *P. hybridus* subsp. ochroleucus demonstrated antioxidant activity (Table 2), with the activity of the leaves’ essential oils greater than that of the rhizomes. Neither of the two essential oils expressed antimicrobial activity against any of the tested bacterial strains (20 pathogenic bacterial strains, e.g., 10 laboratory reference strains and 10 strains isolated from human wound swabs), and certain bacterial strains even appeared to grow better in the presence of the essential oils (although not statistically significant; data not shown). External (topical) application of the essential oils did not cause skin irritation in any of the participating human volunteers, and all of the experimental Albino Swiss Webster mice survived the acute toxicity trial. 

Results obtained from the screening of the interaction of the essential oils with cholinesterase from pooled human serum are presented in Table 3. Essential oils from the leaves and rhizomes of *P. hybridus* subsp. *ochroleucus* inhibited cholinesterase activity, but to a lesser extent then Neositgmin bromide, a synthetic cholinesterase inhibitor. The inhibition by the leaves’ essential oil (33.24%) was lower than the inhibition by the essential oil from the rhizomes (26.37%).

The anti-inflammatory activity results are presented in Table 4. Essential oil from the rhizome significantly decreased edema thickness at the concentrations of 10% (at 1, 4 and 6 h) and 20% (at 4 and 6 h), and the anti-inflammatory activity increased similarly to the referent indomethacin group. Essential oil from leaves decreased edema thickness only at the concentration of 40%, with high efficiency throughout the examined duration and a high percent of anti-inflammatory activity, similar to the indomethacin group. Lower concentrations of the essential oil from the leaves did not show significant results.

The toxicity test on *D. melanogaster* revealed that the essential oils from both the rhizomes and the leaves is toxic for developing larvae. For both essential oils, significant larvae mortality was noted, starting at the concentration of 0.38% (Fischer’s exact test, *p* < 0.01). Estimated 48 h and 96 h LC50s for the rhizome oil were 3.40% and 3.13%, respectively. By comparison, the leaves’ essential oil was four times more toxic, with estimated 48 h and 96 h LC50s of 0.84% and 0.80%, respectively (Table 5). Prolonged monitoring of pupae (until all individuals reached imago stadium) did not show any further significant increase in mortality at the stage of pupa with rhizome essential oil. Only a few individuals that turned into pupae could not achieve imago stadium. However, for the leaves’ essential oil, prolonged mortality was evident during the pupa stage. In the two highest concentrations tested, for both oil types, it was also noted that the larvae grew slower, and both the larvae pupae, and adults were visibly smaller compared to the other groups. 

**Figure 1:**
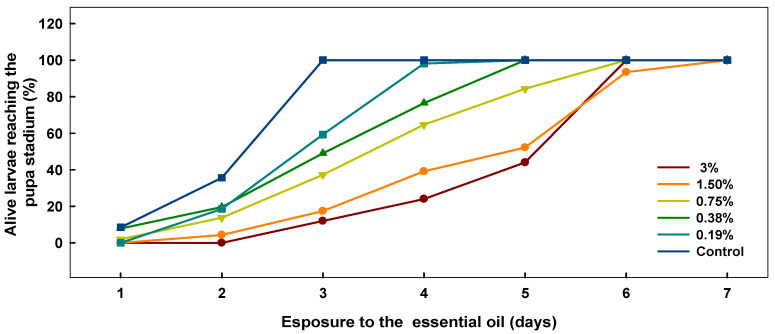
The effect of essential oil from rhizomes of *P. hybridus* subsp. *ochroleucuson* on the development of *D. melanogaster* larvae into pupae. The effect is statically significant for each tested concentration when compared to the control group (Fischer’s exact test *p* < 0.05).

The rhizome oil also affected the development of *D. melanogaster* larvae, and significantly delayed achievement of pupa stadium (Figure 1). The effect was dose-dependent and highly significant when compared to the control (ANOVA overall effect *p* < 0.001 with significant Dunnett’s procedure, for concentrations of 0.75% and higher; chi-square test overall effect *p* < 0.0001 with Fischer’s exact test for each concentration vs. control *p* << 0.05). Development time is presented in Table 5. Compared to the control group, development time for the rhizome essential oil treatment was delayed by 24–60 h depending on the concentration used. In the case of the leaf essential oil, it was not always possible to estimate the development time due to the high mortalities. At the lower concentrations, however, the trend of a delayed pupa stadium was the same as that for the rhizome essential oil.

## 3. Discussion

Plants of the genus *Petasites* contain large amounts of active compounds such as sesquiterpene esters, sesquiterpene lactones and pyrrolizidine alkaloids [3]. Pyrrolizidine alkaloids are toxic [3,4], while some of the sesquiterpene esters and sesquiterpene lactones have medicinal properties. Primarily petasin and isopetasin sesquiterpene esters are considered as the most valuable pharmaceutical components in *Petasites* plants [3]. Therefore, *P. hybridus* plants used for pharmaceutical purposes, and especially the preparations made from these plants, should ideally contain high amounts of petasin/isopetasin, and, most importantly, low amounts of pyrrolizidine alkaloids [17,19]. The chemical analysis of the essential oils of *P. hybridus* ssp. ochroleucus from the Balkans revealed low concentrations of isopetasin, no petasin and no pyrrolizidine alkaloids. Concentrations of sesquiterpene lactones were, however, high. Isopetasin was present only in the rhizome essential oil (3.9%) and absent from the essential oil of the leaves. This concentration is below the average petasin/isopetasin concentration found in other preparations of *Petasites* plants [3]. The distribution and the concentration of petasin/isopetasin is often higher in rhizomes than in leaves [19], which is in accordance with the present results. Lower concentrations of sesquiterpenes (for example, farnesene, bisabolene, cyperene, santalene and germacrene, as well as many others) were detected in the rhizome essential oil, compared to the leaves. All the well-known pyrrolizidine alkaloids commonly present in *Petasites* (senecionine, integerrimine, seneciphylline or senkirkine) [3], as well as other pyrrolizidine alkaloids common in other genera [20], were below the detection limit in the essential oils. This is in accord with pyrrolizidine alkaloids being non-volatiles, which was the drive for the selection of essential oils (that are produced by distillation) as the medium of study. Therefore, essential oils of *P. hybridus* subsp. ochroleucus from the Balkans are of low toxicity, and satisfy the pharmaceutical requirement for low pyrrolizidine alkaloids content. However, as discussed above, they contain only moderate concentrations of the pharmaceutically active petasin/isopetasin, and their medicinal properties are therefore based on the activity of other compounds. 

Absence of pyrrolizidine alkaloids in the essential oils is in accordance with the zero mortality rate observed, and the lack of behavioral changes, in the albino Swiss Webster mice in the acute toxicity trial, as well as with the lack of any adverse reaction following contact with human skin, as demonstrated with the skin irritation assay. The observed toxicity in *Drosophila* flies is therefore related to the presence of various terpene and terpene-like compounds. Plants produce many terpenes as a natural defense against insects [21]. The essential oil of the *P. hybridus* subsp. ochroleucus leaf, which was four times more toxic to *Drosophila* than the rhizome essential oil was, contained high concentrations of linalool (9.03%) (Table 1), which is used as a fruit fly and mosquito insecticide [22,23]. Given that the 48 h LC50 for *Drosophila* larvae was 0.84% (Table 5), and that additional prolonged toxicity hindered pupae ability to reach imago stadium, essential oil from the leaves of *P. hybridus* subsp. ochroleucus can be considered as an efficient insect repellent. 

The anti-inflammatory activity of the essential oils of *P. hybridus*, and the role of petasin/isopetasin, have already been described [24]. In this paper, we examined the anti-inflammatory activity of essential oils obtained from rhizomes and leaves on the Carrageenan-induced paw edema model in rats. Anti-inflammatory activity of oil obtained from rhizome, starting from the concentration of 10%, is high and immediate, while concentration of 20% leads to significant inhibition of edema only after 4 h and repeated applications (Table 4), probably due to the slower dermal absorption of higher concentrations and the accompanying slower effects. Although low, the concentration of the sesquiterpene ester, isopetasine, in the essential oil obtained from the rhizome (3.93%) showed an anti-inflammatory effect after local treatment. The local application mode to the inflammation site probably contributed to the anti-inflammatory activity, because it has been shown that presence of petasin/isopetasin below 15% is inefficient for medical use. Rhizome essential oil at the concentration of 40% did not show significant reduction in paw thickness, which can be associated with problem of dermal absorption of high concentrations of this oil, or an inadequately effective dose. Unlike the essential oil from the rhizome, essential oil from the leaves was effective in suppressing paw edema only at the higher concentrations (statistical significance was obtained at the concentration of 40%). The differences in the effects of the essential oils obtained from rhizomes and leaves are due to different groups of active compounds present in those two plant organs. Sesquiterpenes lactones, which are present at high concentration in leaves’ essential oils (fukinanolide, 33.42%), have some medical properties as well [25], so the absence of petasin/isopetasin in leaves’ essential oils may be offset by high concentrations of sesquiterpenes lactones. This explains the anti-inflammatory effect of the essential oil from the leaves, as well as the higher concentration required to obtain the corresponding response. The efficiency of high concentrations may also indicate better dermal absorption of the essential oil obtained from the leaves.

Essential oil produced by the leaves had a higher antioxidative activity compared to essential oils from the underground parts of the plant, likely due to different chemical composition. Our results for the antioxidant activity of the leaves’ essential oil are supported by results for *Petasites albus*, a different species in the Petasites genus, from Iran [26], which also reported moderate antioxidant activity for the essential oil of the aerial part of the plant. The essential oil obtained from the leaves of *P. hybridus* subsp. ochroleucus contained high concentrations of sesquiterpene lactones (fukinanolide 33.42%) (Table 1). Sesquiterpene lactones of different *Petasites* species are known to have antioxidant activities [25,27,28,29], therefore it is likely that the higher antioxidant activity of the leaves’ vs the rhizome’s essential oil is due to the higher fukinanolide concentration.

Essential oils from the leaves and rhizomes of *P. hybridus* had a moderate anti-cholinesterase activity, with 33.24% and 26.37% inhibition for leaves and rhizomes essential oil, respectively (Table 3). Cholinesterase inhibitors are considered to delay the progression of dementia, and for now only several such inhibitors are officially registered. There is therefore a strong interest in identifying such compounds. Species from the Petasites genus are traditionally used for the treatment of migraine, which suggested their potential for the treatment of other neurological disorders including Alzheimer’s disease e.g., acting as acetylcholinesterase and butyrylcholinesterase inhibitors. The results indeed demonstrate an anticholinergic activity of the tested essential oils, and therefore their a potential value for the the treatment of neurological disorders, acting as AChE and BuChE inhibitors.

## 4. Materials and Methods 

### 4.1. Plant Material

*Petasites hybridus* subsp. ochroleucus, (a subspecies of *Petasites hybridus*) was the plant material studied in the project. This subspecies is spread mainly in the eastern, central and southern parts of the Balkan Peninsula [1,16], and is endemic to the southern Balkan region. It is distinguished from the more common and widely distributed, red-flowered subspecies *P. hybridus* subsp. hybridus, which is related more to the northern and western parts of the Balkans, as well as to the rest of Europe.

Leaves and rhizomes of *P. hybridus* subsp. ochroleucus were collected in September 2014 from natural habitats, close to the village of Petačinci (42°52′50 N; 22°40′19.17 E), Dimitrovgrad, southeastern Serbia. Voucher specimens were deposited in the “Herbarium Moesiacum Niš” (HMN) of the Department of Biology and Ecology, Faculty of Science and Mathematics, University of Niš under the acquisition number 10,800. The official name of the species and status has been checked with http://www.theplantlist.org on 20 October 2017.

### 4.2. Essential-Oil Extraction and Chemical Analyses

The essential oil was extracted from fresh rhizomes and leaves by hydrodistillation for 4 h using a Clevenger-type apparatus. The extracted oil was dried over anhydrous sodium sulfate and stored at 4 °C until further use. Chemical profile of the essential oils was obtained by GC and GC-MS analyses. The GC analysis was performed on a GC HP-5890 II apparatus, equipped with a split–splitless injector, an HP-5MS capillary column (30 m × 0.25 mm, 0.25 μm film thickness) using helium as the carrier gas (1 mL/min), and a flame ionization detector. Operating conditions were as previously reported [30].

Comparison of Kovats retention indices [applying calibrated automated mass spectral deconvolution and identification system software (AMDIS ver. 2.64)], in combination with the SIA resolution method [31], was used for detection of the compounds. Spectral data were compared with the available literature [32], while obtained mass spectra were compared to those from Wiley 275 and NIST/NBS libraries using various search engines. The retention indices were obtained by co-injection with a standard aliphatic hydrocarbons C7–C40 mixture.

Essential oils from leaves and rhizome are complex systems containing numerous compounds, as is shown in Figure S1 (supplemental). Occurrence of the overlapped and embedded peaks is evident in the total ion chromatogram (TIC), as shown in the result of gas chromatography-mass spectrometry (GC-MS) analysis, especially with regard to teropenoids/sesquiterpenoids (compounds 16–62). First, the peaks from TICs obtained by GC-MS were identified by applying SIA. AMDIS was used for the deconvolution of these peaks and extraction of characteristic ion peaks originating from the specific compounds. After deconvolution, the purified mass spectrum of each peak was compared with the National Institute of Standard and Technology (NIST) 08 database or the mass spectra of standards. 

Three examples of this procedure are presented for compounds No. 54, 61 and 62 (Figures S1–S3).

### 4.3. Antioxidant Activity of the Essential Oils 

Antioxidant activity of the essential oils from leaves and rhizomes was tested by DPPH (2,2-diphenyl-1-picrylhydrazyl) and ABTS [2,2′-azino-bis(3-ethylbenzthiazoline-6-sulphonic acid)] assays, as previously described [30], using a Shimadzu, UV-Visible PC 1650 spectrophotometer (Tokyo, Japan). Data analysis was performed with OriginPro 8.0 software (Northampton, MA, USA).

### 4.4. Antimicrobial Activity of the Essential Oils

The antimicrobial activity of the essential oils extracted from *P. hybridus* subsp. ochroleucus was evaluated against 20 pathogenic bacterial strains: (1) A total of 10 laboratory reference strains from the American Type Culture Collection (ATCC)—*Staphylococcus aureus* ATCC 6538, *S. epidermidis* ATCC 12228, *Streptococcus pyogenes* ATCC 19615, *Enterococcus faecalis* ATCC 19433, *Propionibacterium acnae* ATCC 11827 [from group of Gram-positive bacteria], *Escherichia coli* ATCC 9863, *Pseudomonas aeruginosa* ATCC 9027, *Acinetobacter boumanii* ATCC 196060, *Proteus mirabilis* ATCC 12453 and *Klebsiella pneumoniae* ATCC 10031 (from group of Gram-negative bacteria); and (2) A total of 10 bacterial strains isolated from human wound swabs—*Staphylococcus aureus*, *S. epidermidis*, *Streptococcus pyogenes*, *Enterococcus faecalis*, *Propionibacterium acnae*, *Escherichia coli*, *Pseudomonas aeruginosa*, *Acinetobacter sp.*, *Proteus mirabils* and *Klebsiella sp.* (Sorce: Institute of Public Health in Novi Sad, Serbia).

The antibacterial activity of the essential oils against the selected bacteria was evaluated in vitro, by Micro-Well Dilution Assay [33], as was previously described [30]. All experiments were performed in triplicates, and two growth controls, consisting of the corresponding medium with 10% DMSO (as a negative control) and antibiotic Doxycycline (as a positive control), were included (the concentration of Doxycycline ranged from 0.005 to 10.0 mg/mL). Bacterial growth was determined by adding 20 μL of 0.5% triphenyl tetrazolium chloride (TTC) aqueous solution to the plate [34]. Minimal inhibitory concentration (MIC) was defined as the lowest concentration of the oil that inhibited visible growth (red colored pellet on the bottom of the wells after the addition of TTC). For the determination of the minimal bactericidal concentration (MBC), broth was taken from each well that did not exhibit visible growth, and inoculated in Mueller Hinton agar for 24 h at 37 °C. MBC was defined as the lowest oil concentration that killed 99.9% of the bacterial cells. 

### 4.5. Determination of the Skin Irritation Potential of the Essential Oils

The skin irritation potential of *P. hybridus* subsp. ochroleucus oil was determined in 30 male and female healthy volunteers (aged 18–30 years) who showed no signs of dermatological diseases. All volunteers signed an informed consent form after having received a full explanation of the test objectives, procedures and foreseeable risks to subjects. A piece of filter paper saturated (30 µL/cm^2^) either with undiluted *P. hybridus* oils or aqueous solution of sodium lauryl sulfate (20% *w*/*v*, positive control substance) was applied on the skin of the antecubital area of the arm and tightly covered by surgical tape. After 4 h of exposure, the patches were removed, and the sites of application were gently washed with water. The skin was examined and scored at 24, 48 and 72 h after patch removal. Skin reactions were scored, as previously suggested [35], by using a four-point scale. 

### 4.6. Acute Toxicity

Albino Swiss Webster mice of either sex (males, 39–46 g; and females, 31–35 g) from the Oswaldo Cruz Foundation breeding stock were used for the study. Mice were housed, cared and handled in accordance with the Faculty of Medicine, University of Niš-approved guidelines and regulations from 03.07.2007, directed by the government of Serbia, Republic of Serbia Official Gazette No. 37/91, 50/92, 33/93, 52/93, 53/95, 52/96, 48/94, and 25/2000 (health protection of animals). The animals were separated by sex and housed as is described by Mihajilov-Krstev (2014) [30]. All mice had free access to tap water and were fed ad libitum. A single dose of oil of *P. hybridus* obtained from leaves and rhizome, diluted in corn oil, was given by gavages to male (0, 1250, 1500 and 2000 mg/kg body weight) and female (0, 1250, 1500, 2000, 2500 and 5000 mg/kg body weight) mice. An untreated control group was included as well. The mice were observed over 14 days for signs of toxicity or mortality. The procedure was repeated, for the oils extracted from both the underground and the aerial plant parts, separately.

### 4.7. Anti-Cholinesterase Activity

Inhibition of human serum cholinesterase was measured spectrophotometrically using a Konelab 20 analyzer (Thermofisher Scientific, Helsinki, Finland) with flow thermostated cells, length 7 mm (at 405 nm wavelength). Anti-cholinesterase activity was measured as described before [36]. Butyrylthiocholine iodide (purity > 99%), dithio-nitro benzoic acid (DTNB) and neostygmine bromide were purchased from Sigma Co., St. Louis, Missouri, USA. All other chemicals and reagents used [NaH_2_PO_4_-Na_2_HPO_4,_ butylhydroxytoluene (BHT), dimethilsulphoxid (DMSO)] were purchased from Merck, Darmstadt, Germany.

### 4.8. Anti-Inflammatory Activity: Carrageenan-Induced Paw Edema

Anti-inflammation activity of the essential oils was determined on Carrageenan-induced paw edema in Sprague Dawley rats, following Amdekar et al. (2012) [37,38]. Female Sprague Dawley rats (180–250 g) were divided into nine groups of five animals per group. In all groups, 0.1 mL, 1% *w*/*v* λ-carrageenan analytical grade (Sigma-Aldrich Chemie Gmbh., Munich, Germany) in normal saline was injected into the subplantar tissue of the right hind paw. Plant essential oils, obtained from rhizomes and leaves, were dissolved separately in 2.5% DMSO (final volume 50 µL) and applied on the right hind paw of rats from the test groups, in concentrations of 10%, 20% or 40%. Oil was applied twice: first, immediately after the carrageenan injection, and again 3 hours later. The same volume (50 µL) of DMSO was applied to the right paw of animals from the control group. Paw thickness was measured before the carrageenan injection and 1, 2, 4 and 6 h after, using a digital caliper.

Edema thickness (mm) represents increases in the paw thickness at different time intervals after the carrageenan injection, relative to the values obtained before injection. Anti-inflammatory activity (edema inhibition) was calculated as anti-inflammatory activity (%) = (C–T)/(C) ×100, where C represents edema thickness in the control group, and T is the edema thickness in the test group. Indomethacin (10 mg/kg, p.o.) dissolved in saline was used as a standard anti-inflammatory drug, and its anti-inflammatory activity was measured as compared with the control group of animals that received the same amount of normal saline. Indomethacin and saline were given orally, 1 h before the carrageenan was injected. Data is expressed as mean ± S.D. The data was subjected to one–way analysis of variance (ANOVA) followed by Dunnett’s test. Differences between two means were detected by Student *t*-test. Data were considered significantly different for *p* < 0.05.

### 4.9. Drosophila Toxicity Tests

Larvae of *Drosophila melanogaster* Oregon-RC (wild-type flies, stock no. 5) ("Bloomington *Drosophila* Stock Center", Indiana University, USA) fruit flies were used for toxicity testing. Fruit flies were cared for, grown, fed, mated and transferred to the treatment media as previously described [30]. Larvae aged 72 ± 4 h were washed with distilled water and transferred to the standard feeding media containing 0, 0.19, 0.38, 0.75, 1.5 or 3% *v/v* of either rhizome or leaf oil extract from *P. hybridus*. Each experimental/control group consisted of 3 replicas. Each replica contained 20 larvae. Number of pupae, hatched adults and adult mortality were recorded daily. The exposure period lasted until all individuals that were alive emerged from pupae, i.e., 7 and 13 days for the rhizome and leaf essential oil extracts, respectively. As each of the adults emerged from its pupae it was immediately transferred to a new essential oil extract-free feeding medium. Hatched adults were monitored for an additional 7 (rhizome oil group) or 14 (leaf oil group) days after emergence for any prolonged effects on adulthood survival.

Risk Assessment Tool Analysis Software RA V1.0 was used to predict the short term LC50 (the lethal concentration required to kill 50% of the experimental population at a given time) by using surface response model analysis of the results for the *P. hybridus* rhizome and leaf extracts after subchronic exposure. Furthermore, the chronic toxicity threshold (LC1) was derived for the *D. melanogaster* (larva to adult). Development time (larva to pupa; larva to adult) was calculated for each replica of 20 eggs (N = 3) according to the following equation: DT = ∑n_d_*d/n_t_, where n_d_ is the number of pupating larvae/emerging flies d days after the eggs were laid, and n_t_ is the total number of individuals pupating/emerging at the end of experiment. Results were analyzed by one-way ANOVA followed by Tukey’s test and Dunnett’s procedure if significant. In addition, frequency analysis was performed by pooling together three replicas (total N = 60 per concentration) and results were expressed as categorical data (1—pass, or 0—fail). A chi-square test was conducted to assess significant differences in the relative mortality or pupation frequencies. If significant, after residual analysis, Fischer’s exact test was used to compare specific 2 × 2 tables and assess differences in the relative frequencies. Only a *p* value of ≤ 0.05 was considered statistically significant.

## 5. Conclusions

Pyrrolizidine alkaloids were not present in the essential oil of *P. hybridus* subsp. ochroleucus; concentrations of sesquiterpene esters were moderate, and sesquiterpene esters were present only in the rhizome’s essential oil, while essential oil from the leaves had high concentrations of sesquiterpene lactones. The essential oils showed no signs of toxicity to mice and no skin irritation potential in humans, and had no antimicrobial properties. However, they were found to have good local anti-inflammatory activity, a potential for use as an efficient insect repellent (leaves’ essential oils) and a potential value for the treatment of neurological disorders, due to their anticholinergic activity.

## Figures and Tables

**Table 1:** Chemical composition of the essential oils of *Petasites hybridus* subsp. ochroleucus extracted by hydrodistillation from rhizome and leaves.

	Component	RT ^a^ (min)	KIL ^b^	KIE ^c^	Leaves (%)	Rhizome (%)
1.	1-Octene	4.458	788	788.9	–	0.84
2.	1-Nonene	7.068	887	890.7	1.92	8.57
3.	(3E)-Hepten-2-one	8.17	927	925.3	–	0.4
4.	Myrcene	10.289	988	988.1	0.1	0.1
5.	1-Decene	10.368	986	990.4	–	0.15
6.	α-Phellandrene	10.908	1002	1005.9	2.46	2.9
7.	o-Cymene	11.561	1022	1024.1	1.48	1.47
8.	(E)-β-Ocimene	12.334	1044	1045.3	0.09	–
9.	Fenchone	13.637	1083	1081.2	–	0.34
10.	Linalool	14.108	1095	1094.2	9.03	0.77
11.	n-Nonanal	14.481	1100	1104.4	0.08	–
12.	p-Menth-3-en-8-ol	16.166	1145	1151.1	–	0.51
13.	(2E)-Nonen-1-al	16.448	1157	1159	0.62	11.23
14.	Thymol methyl ether	18.917	1232	1228.4	0.2	0.1
15.	1-Octen-3-ol-butanoate	20.634	1280	1277.9	–	0.63
16.	Methyl decanoate	22.218	1323	1324.7	–	0.21
17.	α-Cubebene	22.933	1345	1346.2	–	0.23
18.	α-Longipinene	23.063	1350	1350.1	–	0.57
19.	α-Copaene	23.91	1374	1375.7	1.27	0.76
20.	β-Bourbonene	24.166	1387	1383.4	0.79	1.65
21.	β-Cubebene	24.31	1387	1387.7	1.39	3.13
22.	β-Elemene	24.36	1389	1389.2	1.06	–
23.	Cyperene	24.63	1398	1397.5	–	1.08
24.	α-cis-Bergamotene	25.078	1411	1411.5	–	0.71
25.	α-Santalene	25.313	1416	1419	0.66	3.36
26.	α-trans-Bergamotene	25.726	1432	1432.2	0.15	1.46
27.	Aromadendrene	26.094	1439	1443.9	0.18	0.18
28.	epi-β-Santalene	26.184	1445	1446.7	0.27	3.45
29.	α-Himachalene	26.248	1449	1448.8	0.28	–
30.	(E)-β-Farnesene	26.373	1454	1452.8	0.26	1.34
31.	α-Humulene	26.456	1452	1455.4	0.49	1.68
32.	β-Santalene	26.58	1457	1459.4	–	0.72
33.	4,5-di-epi-Aristolochene	26.904	1471	1469.7	0.48	0.28
34.	Germacrene D	27.303	1484	1482.5	4.26	5.01
35.	Eremophilene	27.517	1486	1489.3	4.31	2.35
36.	epi-Cubebol	27.63	1493	1492.8	0.9	0.44
37.	Valencene	27.706	1496	1495.2	0.26	–
38.	α-Muurolene	27.788	1500	1497.9	0.78	–
39.	β-Bisabolene	28.099	1505	1508.2	–	1.61
40.	(Z)-α-Bisabolene	28.178	1506	1510.9	–	0.42
41.	δ-Cadinene	28.395	1522	1518.2	1.26	–
42.	trans-Calamenene	28.492	1521	1521.4	0.37	0.35
43.	α-Cadinene	29.046	1537	1540.1	–	0.91
44.	α-Calacorene	29.217	1544	1545.8	–	0.21
45.	Spathulenol	30.115	1577	1576.1	0.33	–
46.	β-Copaen-4α-ol	30.522	1590	1589.3	0.37	–
47.	Salvial-4(14)-en-1-one	30.65	1594	1594.1	0.18	0.51
48.	β-Atlantol	31.101	1608	1609.8	1.41	0.3
49.	γ-Eudesmol	31.719	1630	1631.8	–	0.6
50.	Cubenol	32.117	1645	1645.8	–	1.25
51.	α-Eudesmol	32.328	1652	1653.3	–	4.52
52.	α-Cadinol	32.4	1652	1655.3	–	0.37
53.	Dihydro eudesmol	32.493	1661	1659.1	–	0.8
54.	7-epi-α-Eudesmol	32.552	1662	1661.2	16.14	0.69
55.	(E)-Bisabol-11-ol	32.645	1667	1664.5	0.73	0.46
56.	β-Atlantone	32.765	1668	1668.7	–	0.27
57.	β-Bisabolol	32.878	1674	1672.8	–	2.53
58.	(Z)-β-Santalol	34.034	1715	1714.5	0.81	0.67
59.	α-Bisabolol oxide	35.023	1748	1751.5	–	3.38
60.	α-Sinensal	35.111	1755	1754.8	–	1.17
61.	Isopetasin	36.49	1805	1806.6	–	3.93
62.	Fukinanolide	36.949	1824	1824.5	33.42	–
63.	n-Hexadecanol	38.327	1874	1878.2	0.17	–
64.	Palmitic acid	40.532	1959	1967.7	–	1.98
65.	n-Heneicosane	43.801	2100	2106.4	0.22	–
66.	Linoleic acid	44.301	2132	2129.7	–	2.14
67.	Oleic acid	44.691	2141	2147	–	2.62
68.	Larixol	47.191	2265	2260.2	–	0.25
69.	Sempervirol	47.708	2282	2284	–	0.54
70.	4-epi-Abietol	49.06	2343	2348.8	0.49	0.64
71.	Dehydro abietol	49.447	2368	2367.7	0.29	–
72.	cis-Ferruginol acetate	50.288	2411	2408.9	4.25	2.88
73.	6-keto-Ferruginol	51.216	2456	2455.6	0.41	0.35
	**Total**				**94.62**	**92.97**

^a^ RT = Retention time; ^b^ KIL = Kovats (retention) index—literature data; ^c^ KIE = Kovats (retention) index experimentally determined on HP-5MS column.

**Table 2:** Antioxidant activity of the essential oils obtained from rhizomes and leaves of *Petasites hybridus* subsp. *ochroleucus*.

Sample	DPPH IC_50_ (mg/mL)	ABTS mg VitC/g
Essential oil from rhizomes	154.229 ± 0.008	0.082 ± 0.003
Essential oil from leaves	79.899 ± 0.066	1.255 ± 0.043
BHA (0.10 mg/mL)	0.093 ± 0.000	2.660 ± 0.005
Vitamin C (0.10 mg/mL)	0.054 ± 0.001	^ND^

Presented data are averages ± standard deviation (*n* = 4). Lower IC_50_ values and higher ABTS values indicate higher antioxidant activity. The synthetic antioxidant Butylated hydroxyanisole (BHA) and Vitamin C were used as controls. ND: not detected.

**Table 3:** Anti-cholinesterase activity of essential oils obtained from rhizome and leaves of *P. hybridus* subsp. *ochroleucus.*

Sample	% Inhibition/Activation
Essential oil from rhizomes	−33.24
Essential oil from leaves	−26.37
Neostigmin bromide	−96.60

100 µL of essential oil was diluted with DMSO in the ratio 1:5, making the concentration of the essential oil 0.167 µL/mL. Presented results are % of inhibition/activation = [(I_x_ − I_0_)/I_0_] × 100; where I_0_ is activity of serum cholinesterase in human blood serum and I_x_ is activity of human serum cholinesterase in the presence of the tested substances, potential inhibitors or activators.

**Table 4:** Anti-inflammatory activity (in %) of essential oils obtained from rhizome and leaves of *P. hybridus* subsp. ochroleucus.

Groups		Hours			
		1 h	2 h	4 h	6 h
Control (saline)	Edema thickness (mm)	2.56 ± 0.84	2.62 ± 0.61	1.99 ± 0.22	2.49 ± 0.366
	Anti-inflammatory activity (%)	/	/	/	/
Indomethacin (10 mg/kg)	Edema thickness (mm)	1.30 ± 0.32 ^b^	1.45 ± 0.36 ^b^	0.92 ± 0.55 ^b^	1.15 ± 0.183 ^b^
	Anti-inflammatory activity (%)	49.22%	44.65%	53.76%	53.81%
Control (DMSO)	Edema thickness (mm)	2.21 ± 0.80	2.05 ± 0.7	1.98 ± 0.49	2.08 ± 0.42
	Anti-inflammatory activity (%)	/	/	/	/
Rhizome 10%	Edema thickness (mm)	1.10 ± 0.48 ^a^	1.19 ± 0.68	1.07 ± 0.54 ^a^	1.19 ± 0.45 ^a^
	Anti-inflammatory activity (%)	50.22%	41.95%	45.95%	42.7%
Rhizome 20%	Edema thickness (mm)	1.53 ± 0.04	1.34 ± 0.39	1.06 ± 0.34 ^a^	1.16 ± 0.26 ^a^
	Anti-inflammatory activity (%)	30.7%	34.63%	46.46%	44.24%
Rhizome 40%	Edema thickness (mm)	1.79 ± 0.66	1.31 ± 0.74	1.64 ± 0.48	1.76 ± 0.41
	Anti-inflammatory activity (%)	19%	36.09%	17.17%	15.22%
Leaf 10%	Edema thickness (mm)	2.03 ± 0.77	1.51 ± 0.34	1.57 ± 0.47	1.84 ± 0.43
	Anti-inflammatory activity (%)	8.14%	26.34%	20.7%	11.53%
Leaf 20%	Edema thickness (mm)	1.28 ± 0.43	1.24 ± 0.31	1.26 ± 0.56	1.7 ± 0.36
	Anti-inflammatory activity (%)	42.08%	39.51%	36.3%	18.26%
Leaf 40%	Edema thickness (mm)	1.09 ± 0.55	1.15 ± 0.35 ^a^	1.01 ± 0.55 ^a^	1.19 ± 0.57 ^a^
	Anti-inflammatory activity (%)	50.67%	43.9%	48.98%	42.78%

Data are expressed as Mean ± S.D: ^a,b^ *p* < 0.05. ^a^ significant difference between experimental group and control (DMSO) group. ^b^ significant difference between indomethacin group and control (saline) group.

**Table 5:** Effects of essential oils of *Petasites hybridus* subsp. *ochroleucus* obtained from rhizomes and leaves on *D. melanogaster* survival.

	Essential Oil from Rhizomes					
Estimated 48 h LC50 ± SEM for larvae in *w*/*v* %. 95% CI in brackets	3.40 ± 0.34 (2.72–4.06)					
Estimated 96 h LC50 ± SEM for larvae in *w*/*v* %. 95% CI in brackets	3.13 ± 0.50 (2.14–4.11)					
Estimated chronic toxicity threshold: LC1 (larva to adult). 95% CI in brackets.	0.03 (N.A.−0.2)					
Concentration in the feed media (*w*/*v* %)	control	0.19%	0.38%	0.75%	1.5%	3%
Exposed larvae reaching pupa stadium (%)	98.3	90.0	85.0	85.0	76.7	41.7
Exposed larvae reaching imago stadium (%)	98.3	90.0	78.3	83.3	71.7	35.0
DT larva to pupa (days) ± SEM	2.56 ± 0.08	3.25 ± 0.13	3.50 ± 0.37	3.95 ± 0.34	5.02 ± 0.36	5.17 ± 0.46
DT larva to adult (days) ± SEM	7.10 ± 0.03	7.81 ± 0.18	8.25 ± 0.58	9.16 ± 0.20	9.85 ± 0.21	10.40 ± 0.56
	**Essential Oil from Leaves**					
Estimated 48 h LC50 ± SEM for larvae in *w*/*v* %. 95% CI in brackets	0.84 ± 0.40 (0.06–1.62)					
Estimated 96 LC50 ± SEM for larvae in *w*/*v* %. 95% CI in brackets	0.80 ± 0.24 (0.32–1.27)					
Estimated chronic toxicity threshold: LC1 (larva to adult). 95% CI in brackets.	0.07 (0.04–0.10)					
Concentration in feed media in *w*/*v* %	control	0.19%	0.38%	0.75%	1.5%	3%
Exposed larvae reaching pupa stadium (%)	95	93.3	78.3	36.7	11.7	6.7
Exposed larvae reaching imago stadium (%)	95	83.3	68.3	21.7	5.0	1.7
DT larva to pupa (days) ± SEM	1.51 ± 0.03	2.41 ± 0.26	6.20 ± 0.34	4.79 ± 1.09	N.A. *	N.A. *
DT larva to adult (days) ± SEM	6.23 ± 0.14	6.95 ± 0.19	10.57 ± 0.66	N.A. *	N.A. *	N.A. *

* DT is Development time. Where survival rates were < 25% of the number of individuals in the experimental group, DT was not calculated. LC1 is - the toxicity threshold of the toxins concentration.

## Supplementary Materials

The following are available online at https://www.mdpi.com/2223-7747/9/6/700/s1, Figure S1. TIC Chromatogram of rhizome essential oil (Numbers above the peaks corresponding to the numbers in Table 1, Figure S2: Extracted spectrum for the compound No. 54, identified as 7-epi-α-Eudesmol, Figure S3: Extracted spectrum for the compound No. 60, identified as Isopetasine, Figure S4: Extracted spectrum for the compound No. 61, identified as Fukinanolide.
