# Supplementary material for: Phytochemistry, Toxicology and Therapeutic Value of Petasites hybridus Subsp. Ochroleucus (Common Butterbur) from the Balkans
Source: Plants (Basel). 2020 May 31;9(6):700. doi: 10.3390/plants9060700 (PMC7356871; doi:10.3390/plants9060700)
Supplement: Supplementary file 1 [file plants-09-00700-s001.pdf]

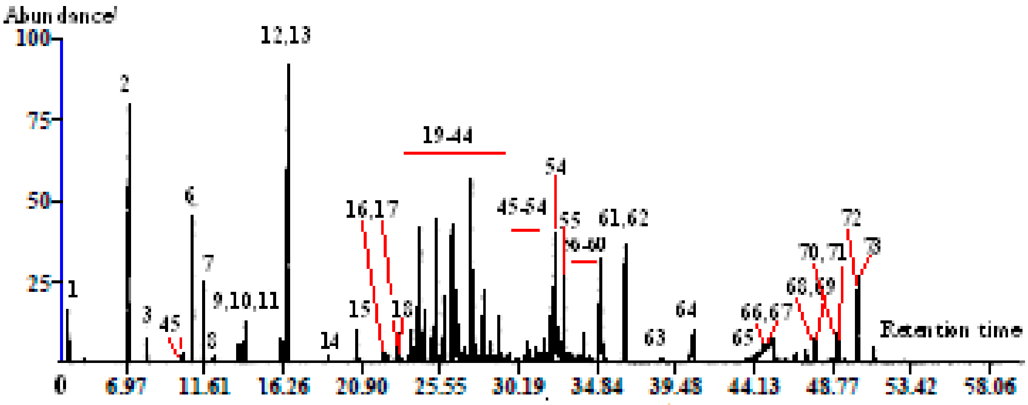

Figure S1. TIC Chromatogram of rhizome essential oil (Numbers above the peaks corresponding to the numbers in Table 1.

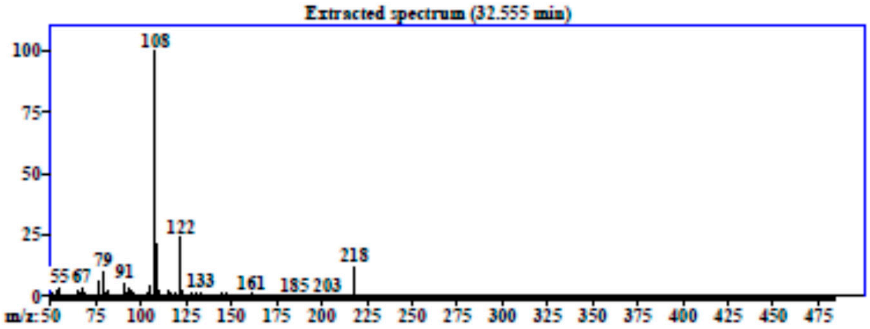

Figure S2. : Extracted spectrum for the compound No. 54, identified as 7-epi-α-Eudesmol.

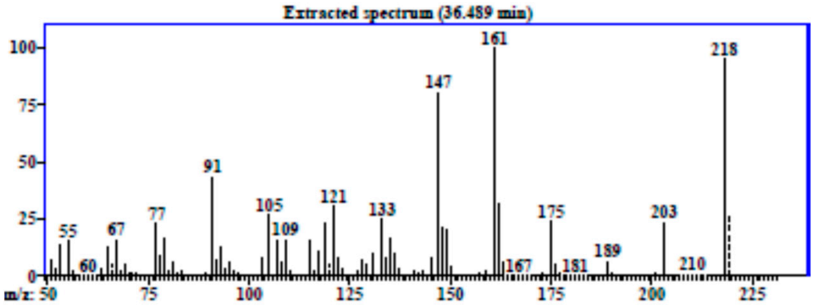

Figure S3.: Extracted spectrum for the compound No. 60, identified as Isopetasine. .

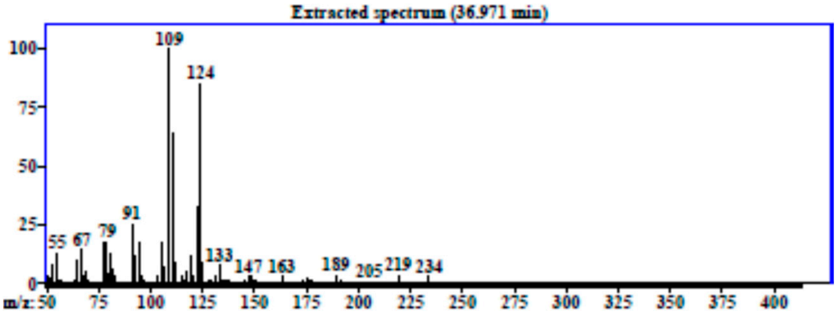

Figure S4.: Extracted spectrum for the compound No. 61, identified as Fukinanolide.
